# Phylogeographic Distribution of Human and Hare *Francisella Tularensis* Subsp. *Holarctica* Strains in the Netherlands and Its Pathology in European Brown Hares *(Lepus Europaeus)*

**DOI:** 10.3389/fcimb.2019.00011

**Published:** 2019-02-11

**Authors:** Miriam Koene, Jolianne Rijks, Miriam Maas, Robin Ruuls, Marc Engelsma, Peter van Tulden, Marja Kik, Jooske IJzer, Daan Notermans, Maaike de Vries, Ewout Fanoy, Roan Pijnacker, Marcel Spierenburg, Herjan Bavelaar, Hanneke Berkhout, Sanjay Sankatsing, Rob Diepersloot, Kerstin Myrtennas, Malin Granberg, Mats Forsman, Hendrik-Jan Roest, Andrea Gröne

**Affiliations:** ^1^Department of Bacteriology and Epidemiology, Wageningen Bioveterinary Research, Wageningen University and Research, Lelystad, Netherlands; ^2^Faculty of Veterinary Medicine, Dutch Wildlife Health Centre, Utrecht University, Utrecht, Netherlands; ^3^National Institute for Public Health and the Environment (RIVM), Bilthoven, Netherlands; ^4^GGD Rotterdam Rijnmond, Rotterdam, Netherlands; ^5^Netherlands Food and Consumer Product Safety Authority, Utrecht, Netherlands; ^6^Department of Medical Microbiology and Infectious Diseases, Canisius Wilhelmina Hospital, Nijmegen, Netherlands; ^7^Department of Internal Medicine, Diakonessenhuis, Utrecht, Netherlands; ^8^Department of Medical Microbiology en Immunology, St. Antonius Hospital, Nieuwegein, Netherlands; ^9^Swedish Defence Research Agency, Umeå, Sweden

**Keywords:** *Francisella tularensis* subspecies *holarctica*, tularemia, Netherlands, genotyping, human, European brown hare (*Lepus europaeus*), pathology

## Abstract

Sequence-based typing of *Francisella tularensis* has led to insights in the evolutionary developments of tularemia. In Europe, two major basal clades of *F. tularensis* subsp. *holarctica* exist, with a distinct geographical distribution. Basal clade B.6 is primarily found in Western Europe, while basal clade B.12 occurs predominantly in the central and eastern parts of Europe. There are indications that tularemia is geographically expanding and that strains from the two clades might differ in pathogenicity, with basal clade B.6 strains being potentially more virulent than basal clade B.12. This study provides information on genotypes detected in the Netherlands during 2011–2017. Data are presented for seven autochthonous human cases and for 29 European brown hares (*Lepus europaeus*) with laboratory confirmed tularemia. Associated disease patterns are described for 25 European brown hares which underwent post-mortem examination. The basal clades B.6 and B.12 are present both in humans and in European brown hares in the Netherlands, with a patchy geographical distribution. For both genotypes the main pathological findings in hares associated with tularemia were severe (sub)acute necrotizing hepatitis and splenitis as well as necrotizing lesions and hemorrhages in several other organs. Pneumonia was significantly more common in the B.6 than in the B.12 cases. In conclusion, the two major basal clades present in different parts in Europe are both present in the Netherlands. In hares found dead, both genotypes were associated with severe acute disease affecting multiple organs. Hepatitis and splenitis were common pathological findings in hares infected with either genotype, but pneumonia occurred significantly more frequently in hares infected with the B.6 genotype compared to hares infected with the B.12 genotype.

## Introduction

Tularemia, caused by the bacterium *Francisella tularensis*, may be regarded as a relatively new disease in Western Europe. Until 1929, the disease was restricted to Russia and parts of Scandinavia. In the late 1930s the disease was first reported in (current) Austria, and additional outbreaks were described in following decades from several other countries like Germany, France, and Belgium (Jusatz, 1952). Italy and Spain are the most recent countries to report cases, in 1964 and 1997, respectively (Dwibedi et al., 2016). In Europe, disease is almost exclusively caused by *F. tularensis* subsp. *holarctica*.

Because of the limited genetic variation in *F. tularensis*, single nucleotide polymorphisms (SNP) and multiple loci variable number of tandem repeats analysis (MLVA) are the preferred genetic markers for molecular typing. A first set of canonical SNPs (canSNPs), representing key positions along the *Francisella* phylogenetic tree were identified and labeled by serial numbers (Svensson et al., 2009; Vogler et al., 2009) and the resulting phylogenetic structure is still the basis for the current nomenclature for *F. tularensis* subsp. *holarctica* genotypes; an acronym using the letter “B” for the subspecies *holarctica*, and for subclades the characters “Br.” followed by the branch numbers named for the two flanking canSNPs (for instance B.Br.010/011), or the designated identification of a reference strain (for instance B.Br.FTNF002-00).

Since then, there has been a substantial increase in the available molecular data of *F. tularensis* strains from Europe (Chanturia et al., 2011; Gyuranecz et al., 2012; Karlsson et al., 2013; Afset et al., 2015; Sissonen et al., 2015; Dwibedi et al., 2016; Schulze et al., 2016). Additional serial numbered SNPs have been used to increase the discriminatory power. As a result, the phylogenetic tree for *F. tularensis* subsp. *holarctica* has expanded tremendously (Larkeryd et al., 2014). This inherently led to continuous changes in terminology, sometimes making it difficult to compare results from various studies. Nomenclature has been simplified by removing the “Br.” and instead of using the two flanking canSNPs, only the marker that defines the branch is now specified. More important, with new strains and additional subclades being included, major genetic lineages for subspecies *holarctica* are also distinguished. The current nomenclature defines four basal clades within the subspecies *holarctica*; B.4, B.6, B.12, and B.16. In Europe, most *F. tularensis* subsp. *holarctica* strains belong to basal clades B.6 and B.12.

Basal clade B.6 includes subclades B.7 and B.10. The B.7 subclade (also referred to as genotype B.40 or B.Br.OR96-0246) is predominantly found in Scandinavia (Karlsson et al., 2013; Afset et al., 2015; Sissonen et al., 2015). The B.10 (also designated as B.41) subclade is mainly represented by strains that are members of B.11, previously referred to as the (Franco)Iberian clone (Dempsey et al., 2007). This B.11 subclade is characterized by a 1.59 kb deletion, termed RD23, and matches the B.Br.FTNF002-00 subpopulation as defined by Vogler (Vogler et al., 2009). This B.11 subclade is present in the western part of Europe (Gyuranecz et al., 2012; Dwibedi et al., 2016).

**Figure 1:**
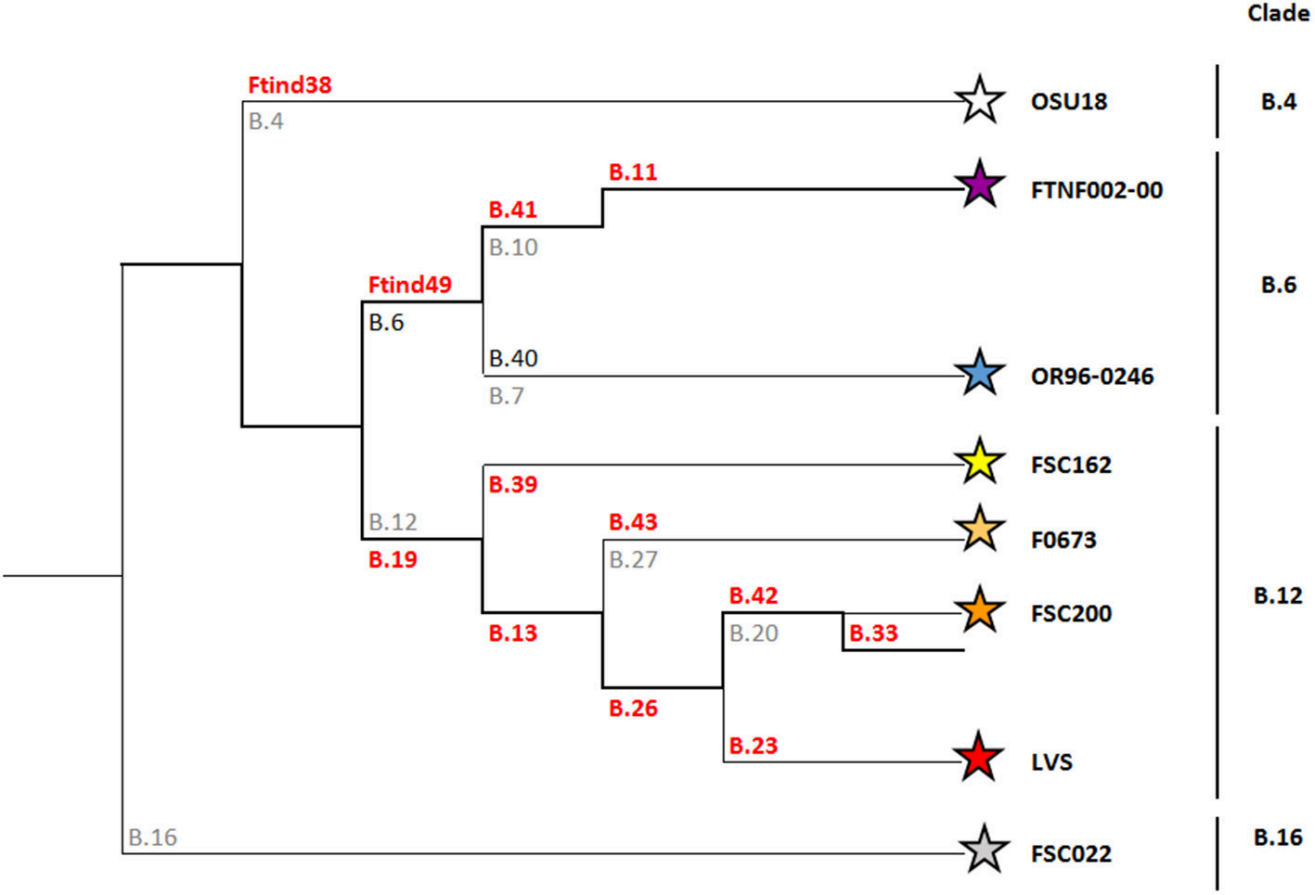
Phylogeny of *Francisella tularensis* subsp. *holarctica*. SNP and Indel markers, for which assays have been developed, are depicted in orange. These markers define the four major canSNP clades B.12, B.6, B.16, and B.4, and six subclades (Karlsson et al., 2013). For guidance, previously published corresponding markers are depicted in gray.

Basal clade B.12 includes subclade B.13 previously described as B.Br.013/014 (Gyuranecz et al., 2012), and can be further divided into subclades B.27 (also known as B.43), B.20 (also known as B.42) and B.23 (Figure 1). A perfect correlation between clade B.12 and Biovar II strains has recently been shown (Karlsson et al., 2016). A unique distinctive feature of Biovar II strains is resistance to erythromycin, oleandomycin and lincomycin (Kudelina and Olsufiev, 1980). Strains belonging to clade B.12 (and Biovar II) are predominantly found in East and Central Europe (Kudelina and Olsufiev, 1980; Gyuranecz et al., 2012; Karlsson et al., 2016).

Genotyping studies have shown that subspecies *holarctica* probably originates from Asia or North America and has successfully disseminated worldwide in recent evolutionary time (Johansson et al., 2004; Keim et al., 2007; Vogler et al., 2009; Petersen and Molins, 2010; Karlsson et al., 2013; Lu et al., 2016). However, the spread of the disease within Europe is poorly understood, as are potential differences in pathology and pathogenic potential between genotypes. Pathology was found to be different in European brown hares (*Lepus europaeus*) infected with the B.6 clade in Switzerland compared with those infected with the B.12 genotype in Switzerland and to the lesions described in Hungary (Gyuranecz et al., 2010; Origgi and Pilo, 2016). These results suggested differences in pathogenesis between the two distinct clades of *F. tularensis* subsp. *holarctica* in Europe. In addition, experimental infection of Fischer 344 rats with a B.6 subclade B.11 strain from Italy resulted in a more severe disease presentation when compared to rats infected with a B.12 genotype from Hungary (Kreizinger et al., 2017).

The first reported case of human tularemia in the Netherlands dates back to 1953, when multiple members of a family contracted tularemia by consumption of a European brown hare that was found dead (Hemmes, 1953). No endemic human tularemia cases were reported in the Netherlands until 2011, when a human tularemia case was identified with no history of traveling abroad (Coolen et al., 2013; Maraha et al., 2013). Subsequently, between 2011 and the end of 2017, a total of 17 laboratory confirmed human cases of indigenous tularemia were identified. Surveillance of tularemia in Dutch European brown hares started in 2011, and the first hare case was confirmed in 2013. A total of 29 hare laboratory confirmed cases were diagnosed throughout the country by the end of 2017 (Rijks et al., 2013; Janse et al., 2017). Additionally, tularemia was identified in primates from a shelter house (Bolhuis et al., 2017) and in water specimens (Janse et al., 2017). It is unclear to what extent disease emergence and/or increased awareness and detection explain the recent increase in human and hare cases in the Netherlands. Genetic information from autochthonous strains and associated disease patterns may provide some clues and is expected to improve the understanding of the epidemiology of the disease.

This paper describes the genotyping results based on a hierarchical real-time PCR typing scheme using several canSNPs and canonical insertion deletion mutations (canINDEL) markers. Genotyping results, clinical presentation and likely route of infection are presented for seven autochthonous human cases in which infection with *F. tularensis* was detected by PCR and/or culture. Additionally, genotyping results from 29 hares are described and pathology findings analyzed per genotype to investigate whether the frequency of occurrence and the severity of lesions differs between clades.

## Materials and Methods

### Cases in Humans

Of the 17 human cases of indigenous tularemia in the period 2011–2017, laboratory confirmation was based on either serology (*n* = 6), detection of F. tularensis (*n* = 6), or both (*n* = 5). For this study genotyping results from seven patients were available (Table 1). DNA was either obtained from isolates (*n* = 3) or directly from wound exudate or suppurative lymph node material submitted for diagnostic testing (*n* = 4). All materials from patients were tested anonymously. The most likely source and location of infection was determined based on patient interviews by local public health services. Ethical approval was not needed according to institutional and national guidelines. It was confirmed by the Utrecht medical ethics committee that the study is exempt from further ethics approval (METC UMC Utrecht, reference number 18-617).

**Table 1:** Human cases of tularemia in the Netherlands included in this study (2011–2017).

**Basal clade**	**Subclade**	**Month/year of infection**	**Most likely location of infection (province)**	**Most likely transmission route**	**Clinical presentation of tularemia**	**Reference (if applicable)**
B.6	B.11^a^	October 2011	Overijssel	Insect bite	Ulceroglandular	Maraha et al., 2013
B.6	B.11	July 2013	Limburg	Insect bite	Ulceroglandular	Leenders et al., 2015
B.6	B.11^b^	August 2015	Utrecht, possibly Overijssel	Insect bite or swimming	Ulceroglandular	
B.6	NT	October 2016	Utrecht	Swimming	Glandular	
B.6	B.11^b^	Dec 2016^*^	Gelderland	Direct contact with an infected hare	Ulceroglandular	
B.12	B.20^c^	Jan 2014^**^	Zeeland	Direct contact with an infected hare	Glandular	
B.12	B.33^b^	May 2016	South Holland	Insect bite	Glandular	

* *One hare linked to a human case. Pathological examination not possible on this specimen because it had been eviscerated and skinned*.

** *Human case linked to a hare. Genotype of the human patient identical to that in the hare linked to this case*.

a *CanSNPer result (Coolen et al., 2013; Maraha et al., 2013)*.

b *CanSNPer identification*.

c *The clade is denoted B.20 since the first canSNP used for this branch was B.20. In this study, B.42 was used for typing which is another canSNP along the same branch. NT, not determined*.

### Cases in Hares

DNA used for genotyping of 29 European brown hare cases was obtained directly from specimen material that had been submitted for diagnostic testing. Of those, 27 confirmed hare cases were submitted in context of the Dutch national wildlife disease scanning surveillance program, and two hares were submitted because they were thought to be the source of human infection through skinning of the hares. In the 27 hares from the surveillance program, the presence of *F. tularensis* genetic material had been confirmed in three tissues (lung, liver and spleen) by a diagnostic PCR-test, as described earlier (Rijks et al., 2013). In the two hares linked to human cases, the specimen materials were muscle and bone marrow, as organs were no longer available at the time of testing. The spatial distributions of the human cases and the hare cases were mapped using ArcGIS^©^ software.

For comparison, significant lesions that were found in the sampled hares were grouped according to the *Francisella* genotype. Case histories and descriptions of lesions had been recorded in necropsy reports; body condition was scored based on degree of fat storage and muscle development, and gross pathology findings were recorded following a standard protocol. For histopathology, tissue specimens were fixed in 4% phosphate-buffered formalin, embedded in paraffin, cut into 4-μm sections, and stained with hematoxylin and eosin. Tissues examined routinely using histopathology were lung, heart, spleen, liver, stomach, duodenum, jejunum, ileum, caecum, colon, kidney, adrenals, and brain. Other tissues were only sampled and examined if there was a clear indication for lesions.

### Genotyping

DNA was obtained from standard specimens that were positive for the presence of *F. tularensis* by TaqMan real-time PCR using the FTT0376 primers and probe published by Mitchell et al. (2010). Specimen material was processed under BSL3 conditions. All containment facilities comply with national and international laws and regulations concerning biosafety (EU Directive 2000/54/EC) and biosecurity (Code of Conduct of the Royal Netherlands Academy of Sciences, KNAW). DNA isolation for PCR testing was performed using either the DNeasy Blood and Tissue Kit (Qiagen, Hilden, Germany), or NUCLISENS^®^ easyMAG^®^ (Biomerieux, France). For the latter, material from swabs was suspended in PBS from which 500 microliter was added to 2 ml lysis buffer. Whenever isolates were available, about 10 microliter from a fresh culture (BSL3) was suspended in 600 microliter lysis buffer, followed by an inactivation step in a heating block at 80°C during 5 min. After the lysis step, the material was further processed under BSL2 conditions in the easyMAG.

For the identification of subclades of *F. tularensis* a hierarchical real-time PCR typing scheme based on binary output of PCR results targeting insertion/deletions and SNPs was used (Larsson et al., 2007; Svensson et al., 2009; Larkeryd et al., 2014). This assay can be used on DNA obtained from clinical samples routinely processed without the need for pure cultures of the bacteria.

In four cases, strains were available for whole genome sequencing. The genotyping of the 2011 patient has been described in detail elsewhere (Coolen et al., 2013; Maraha et al., 2013). For the remaining strains, DNA was isolated using a Gentra Puregene kit (QIAGEN GmbH, Germany), and whole-genome sequencing was performed using TruSeq library based paired-end 250 bp sequencing (Illumina MiSeq, San Diego, CA, USA). Demultiplexing of the data was performed with standard Illumina software from the casave pipeline with default settings. The quality of sequence reads was analyzed using FastQC tool (v0.11.5). All artifacts were polished from the Illumina short reads using the BBMap suite (37.66). Draft *de novo* genome assemblies were created using SPAdes (version 3.10.0; Bankevich et al., 2012) on BBduk adapter-clipped and quality trimmed data (>Q20; BBMap v39.92-Bushnell B. -https://sourceforge.net/projects/bbmap/). Strain typing was performed using CanSNPer on the draft genome assemblies (Larkeryd et al., 2014). Sequence data have been submitted to the European Nucleotide Archive under study number PRJEB27514 and accession numbers ERS2585217, ERS2585218, and ERS2585220 as shown in the Supplementary Table.

### Statistical Analyses

To investigate whether the odds of hepatitis, splenitis, pneumonia or adrenalitis were greater in the B.6 genotype than in the B.12 genotype, one-sided Fisher-exact tests were performed in R (R Core Team, 2013). R: A language and environment for statistical computing. R Foundation for Statistical Computing, Vienna, Austria).

## Results

### Human Cases of Tularemia in the Netherlands

**Figure 2:**
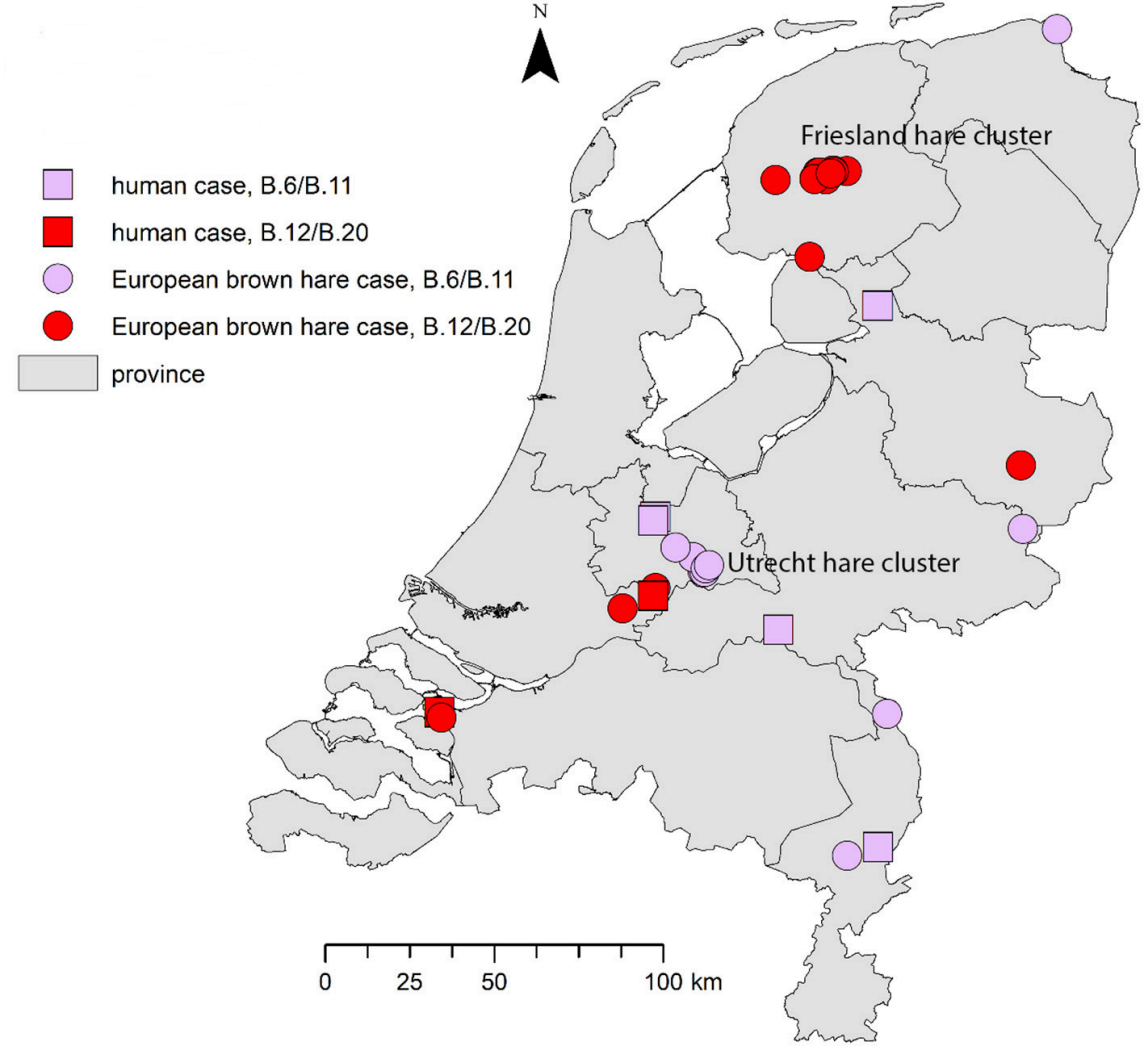
Geographical distribution of tularemia cases in humans and hares. For the human cases the most likely location of infection was determined based on patient interviews by local health authorities. The proven hare-to-human case is shown in the south-west.

Genotyping results for seven reported cases in humans are summarized in Table 1. All patients included in this study presented with the ulceroglandular or glandular form of tularemia. Based on patient interviews, the most likely locations of infection are shown in Figure 2.

*F. tularensis* subsp. *holarctica* strains from five patients were assigned to basal clade B.6, the remaining two cases the strains were assigned to B.12 (Table 1). Four of the B.6 strains were further genotyped to subclade B.11 (also referred to as the Iberian clone; B.Br.FTNF002-00), which is typical for Western Europe. No subtyping was performed for the remaining B.6. strain. The two B.12 strains were shown to be members of subclade B.20 (also called B.42), a canSNP group common in Scandinavia, Central and Eastern Europe (Table 1). In one of the B.12 cases an isolate was available for WGS and using the CanSNPer pipeline was found to belong to B.33 which is a subgroup of B.20.

Two of the seven human tularemia cases described in this paper occurred after direct contact with infected hares. In one case, infection was acquired through insect bites in the same area and period, as where and when an infected hare had been found (Table 1; Leenders et al., 2015).

### European Brown Hare Cases of Tularemia in the Netherlands

Genotyping results of the 29 European brown hares showed that 12 animals were infected with clade B.6 strains and 17 animals with clade B.12 strains (Table 2). For the B.6 cases, one strain could be further assigned to subclade B.11, while in 13 strains of clade B.12 further subtyping to subclade B.20 was achieved.

**Table 2:** Spatiotemporal and host features of European brown hare tularemia cases per *Francisella tularensis* subspecies *holartica* genotype.

**Basal clade**	**Subclade**	**Month/year of infection**	**Location (province)**	**No. of cases**	**Age category^§^**	**Sex^$^**	**Body condition^&^**
B.6	B.11	May 2013	Limburg	1	A	M	G
B.6	NT	June 2015	Gelderland	1	A	M	M
B.6	NT	March 2016	Groningen	1	A	M	P
B.6	NT	May 2016, Oct-Nov 2016, Mar 2017, May 2017	Utrecht	8^*^^#^	5A, 1I	3M, 2F	3G, 1M, 2P
B.6	NT	Nov 2017	Limburg	1	I	F	Moderate
B.12	B.20^a^	Jan 2014	Zeeland	1^**^			
B.12	B.20^a^	April 2014	Utrecht	1	A	M	M
B.12	B.20^a^	Feb-May 2015	Friesland	11	8A, 1I	9M, 2F	2G, 7M, 2P
B.12	B.20^a^	March 2015	Friesland	1^#^			
B.12	B.20^a^	March 2015	Overijssel	1	A	M	G
B.12	NT	Jan 2016	Friesland	1	A	M	M
B.12	NT	Oct 2016	South Holland	1	I	M	M
2 clades		5 years	8 provinces	29 cases			

* *One hare linked to a human case. Pathological examination not possible on this specimen because it had been eviscerated and skinned*.

** *Hare linked to a human case. Pathological examination not possible on this specimen because it had been eviscerated and skinned. Genotype (determined in muscle tissue) identical to that in the human patient linked to this case*.

# *One hare too autolytic for pathological examination*.

§ *Age category (when determined): A, Adult; I, Immature*.

$ *Sex (when determined): M, male; F, female*.

& *Body condition (when determined): G, good; M, moderate; P, poor*.

a *The clade is denoted B.20 since the first canSNP used for this branch was B.20. In this study, B.42 was used for typing which is another canSNP along the same branch*.

Both clades are widespread over the country: the B.6 and B.12 cases have been detected in four and five of the 12 Dutch provinces, respectively. Both genotypes were found in the province of Utrecht (Table 2; Figure 2). A number of the European brown hare cases included in this study were temporally and geographically linked; eight of the B.6 cases were found in a region of 2.5 by 12.5 km in direct distance of a meandering riverbed in the period between May 2016 and May 2017. For B.12, 11 hares were part of an outbreak of tularemia that lasted from February to May 2015 in several adjacent municipalities in the province of Friesland (Janse et al., 2017). Other cases were individual submissions belonging to distinct events in space and time (Table 2; Figure 2).

The main reason why hares were submitted to the surveillance program was the finding of multiple dead hares in a restricted area within a short time frame (nine out of 11 B.6 cases; 14 out of 16 B.12 cases). Other reasons were atypical behavior prior to death, such as unusual movement patterns (e.g., a swaying gait), falling over, excessive digging or a freezing state in between bouts of hopping (*n* = 6); lack of fear (*n* = 5); or death in an unusual place, interpreted by submitters as lack of hiding behavior (*n* = 6). In four events, no other dead hares were observed, but there had been atypical behavior (1/11 B.6; 1/16 B.12), a poor general body condition (1/16 B.12), or on the contrary a surprisingly good general body condition (1/11 B.6). Scavengers such as crows were occasionally reported to have been pecking at the carcasses. No information was available on the history of the two hares linked to the human cases.

Pathological investigations were performed on 25 European brown hares, 10 infected with B.6 and 15 with B.12. In both groups, the submitted animals were mainly adult males, with a moderate to good body condition (Table 2). Poor body condition was observed only in 3/10 (30%) of the B.6 cases and 2/15 (13%) of the B.12 cases (Table 2).

Macroscopically, lungs, trachea, spleen, and liver were the organs most affected (Table 3). In the lungs, hemorrhages, hyperemia, edema, emphysema or a combination of these were noticeable in all B.6 cases and in 11/15 (73%) of the B.12 cases. In both groups, the tracheal mucosa was frequently hyperemic, and the lumen sometimes contained blood clots. The spleen was enlarged in all B.6 cases, and in two-thirds of the B.12 cases. Liver lesions like reduced consistency, enlargement, hyperemia, beige coloring, dullness, or a combination of these were observed in 7/10 (70%) of the B.6 cases, and in 11/14 (78%) of the B.12 cases. Single or multiple white or yellowish foci, ranging in size from 1 to ~7 mm in diameter, were observed in seven hares in various organs: the foci were detected in lungs (2 B.6; 1 B.12), liver (1 B.6; 1 B.12), mediastinum (1 B.12), or abdominal serosa (1 B.6). In both groups, the stomach was generally well or moderately filled with food. Enlarged lymph nodes were not recorded, apart from an enlarged mesenteric lymph node in one B.12 case. Lesions suggestive of trauma, such as broken skin or bones, picked eyes and intracranial or neck hemorrhages were observed in 6/10 (60%) B.6 cases, and 8/15 (53%) B.12 cases. Incidental lesions consisted of poor dentition (*n* = 2), icterus, and a kidney with beige firm areas (amyloidosis).

**Table 3:** Overview of pathology per genotype.

**Organ**	**Gross pathology B.6/B.11**	**Histopathology B.6/B.11**	**Gross pathology B.12/B.20**	**Histopathology B.12/B.20**
Lung	10/10 with lesions: - 6/10 (multi-)focal hemorrhage(s) - 7/10 hyperemia - 6/10 edema - 3/10 emphysema - 2/10 foci (in one case described as yellow, dull, 2x3mm to 9x6mm foci)	10/10 with lesions: - 6/10 with pneumonia: ^*^5/6 necrotizing (*n =* 4) or necrotizing suppurative (*n =* 1) pneumonia, (sub-)acute, generally severe (*n =* 4) ± bacteria (*n =* 3), hemorrhages, hyperemia, edema, emphysema ^*^1/6 suppurative interstitial pneumonia, acute, mild ± bacteria, hyperemia, edema, emphysema - 3/10 hemorrhage ± edema, hyperemia, emphysema - 1/10 only hyperemia, edema, emphysema	5/15 NS - 10/15 with lesions: - 7/10 (multi-)focal hemorrhage(s) - 4/10 hyperemia - 7/10 edema - 1/10 emphysema - 1/10 foci (described as one firm white nodule)	15/15 with lesions: - 2/15 with pneumonia: 15 necrotizing pneumonia ^*^1/15 necrotizing pneumonia, sub-acute, severe with hemorrhages, and hyperemia ^*^1/15 acute mild suppurative interstitial pneumonia with hyperemia, edema - 5/15 hemorrhage ± hyperemia, edema, emphysema - 8/15 only hyperemia, edema, emphysema
Trachea	1/10 NA - 1/9 NS - 8/9 with lesions: - 6/8 hyperemia - 2/8 hemorrhage	8/10 NA - 2/10 with lesions: - 1/2 hemorrhage in serosa - 2/2 hyperemia	5/15 NS 10/15 with lesions: - 7/10 hyperemia - 5/10 hemorrhage - 1/10 edema	15/15 NA
Heart	10/10 NS	9/10 NS - 1/10 with lesions: - 1/1 subendocardial petechia	1/15 ND 12/14 NS 2/14 with lesions: - 1/2 beige-pink color - 1/2 yellow aorta (icterus)	1/15 NA 13/14 NS 1/14 with lesions: - 1/1 aorta hemorrhage
Spleen	10/10 with lesions: - 10/10 splenomegaly	10/10 with lesions: - 9/10 splenitis: ^*^8/9 necrotizing (*n =* 7) or necrotizing suppurative (*n =* 1) splenitis, acute, generally severe (*n =* 6), ±bacteria (*n =* 3), hemorrhages in the red pulp (*n =* 4) ^*^1/9 necrotizing granulomatous splenitis, subacute, severe, with lymphodepletion - 1/10 hyperemia and PALS	5/15 NS - 10/15 with lesions: 10/10 splenomegaly	2/15 NA (autolytic) 13/13 with lesions: - 9/13 necrotizing splenitis, acute, generally severe (*n =* 8), ±bacteria (*n =* 3), hemorrhages in the red pulpa (n = 1), and lymphodepletion (*n =* 4). - 1/13 acute hemorrhages - 3/13 only hyperemia ±hyperplasia white pulp (*n =* 1), PALS (*n =* 2)
Lymph nodes	10/10 NS	7/10 NA 3/10 with lesions: - 3/3 necrotizing lymphadenitis, cervical (*n =* 1), bronchial (*n =* 1), mesenterial (*n =* 1), with bacteria (*n =* 2)	14/15 NS - 1/15 with lesions: - 1/1 enlarged mesenteric lymph nodes	13/15 NA 2/15 with lesions: - 2/2 necrotizing lymphadenitis, bronchial (*n =* 1) and mesenterial (*n =* 2) ln, ± bacteria (*n =* 1), hemorrhages (*n =* 1)
Liver	3/10 NS - 7/10 with lesions: - 5/7 reduced consistency - 3/7 enlargement - 2/7 beige color - 1/7 hyperemia - 1/7 dull (necrosis) - 1/7 foci, widespread light-yellow foci	10/10 with lesions: - 10/10 necrotizing hepatitis, focal (*n =* 1) or multifocal (*n =* 9), (sub)acute, mild (*n =* 3) to severe (*n =* 7)	1/15 ND 3/14 NS 11/14 with lesions: - 3/11 reduced consistency - 3/11 enlargement - 3/11 multifocally beige - 1/11 hyperemia - 1/11 dull (necrosis) - 1/11 foci, multifocal, white, 1-2 mm diameter	15/15 with lesions: - 14/15 necrotizing hepatitis, focal (*n =* 1) or multifocal (*n =* 12), (sub)acute, mild (*n =* 1) or severe (*n =* 13), ±bacteria (*n =* 2) - 1/15 hyperemia
Stomach	9/10 NS 1/10 empty	4/10 NA (autolytic) 6/10 NS	14/15 NS 1/15 empty	2/15 NA (autolytic) 13/15 NS
Intestine	5/10 NS 5/10 with lesions or abnormal contents: - 1/5 hyperemic duodenum with red fluid (blood) in lumen - 1/5 thickened colon wall with red (blood) mucus in lumen - 3/5 beige-brown (mucous) fluid in lumen	2/10 NA (autolytic) 6/8 NS (nematodes *n =* 4) 2/8 with lesions: - 2/2 hemorrhages in colon, serosa (*n =* 1) or mucosa (*n =* 1)	1/15 ND 10/14 NS 4/14 with lesions or abnormal contents: - 1/4 with hyperemic duodenum. - 3/4 remaining cases: brown (mucous) fluid in lumen	3/15 NA (autolytic) 10/12 NS (nematodes *n =* 4; coccidia *n =* 3) 2/12 with lesions: - 1/2 necrosis submucosa colon - 2/2 hemorrhages in colon, serosa (*n =* 1) or mucosa (*n =* 1)
Adrenals	10/10 NS	1/10 NA 3/9 NS 6/9 with lesions: - 4/6 necrotizing adrenalitis, focal (*n =* 1) to multifocal (*n =* 3), mild (*n =* 1) to severe (*n =* 3), subacute (*n =* 1) to acute (*n =* 3) ±bacteria (*n =* 4) - 2/6 hyperemia with bacteria	3/15 ND 12/12 NS	3/15 NA 3/12 NS 9/12 with lesions: - 3/9 necrotizing adrenalitis, focal (*n =* 2) to multifocal (*n =* 1), mild (*n =* 2) to severe (*n =* 1), acute (*n =* 3) ±bacteria (*n =* 1) - 2/9 capsule hemorrhage - 3/9 hyperemia - 3/9 bacteria, no reaction
Kidney	6/10 NS 4/10 with lesions: - 2/4 hyperemia - 1/4 diffusely beige - 1/4 multifocal beige areas (amyloid)	5/10 NS - 5/10 with lesions: - 4/5 hyperemia - 1/5 amyloidosis (incidental finding)	1/15 ND 10/14 NS 4/14 with lesions: - 1/4 hyperemia - 2/4 diffusely beige - 1/4 reduced consistency - 1/4 yellow papil (icterus)	2/15 NA 10/13 NS 3/13 with lesions: - 3/3 hyperemia
Serosa with fat tissue	9/10 NS 1/10 with lesions: - 1/1 multiple 1mm diameter foci in spleen serosa, uterus serosa, mesometrium and abdominal diaphragm.	6/10 NA 4/10 with lesions: 4/4 hemorrhage in serosa/fat surrounding thyroid (*n =* 1), pancreas (*n =* 1), kidney (*n =* 1) gonad (*n =* 1)	14/15 NS 1/15 with lesions - 1/1 with 1 yellowish focus in mediastinum	10/15 NA - 5/15 with lesions: - 2/5 necrotic foci in fat tissue, e.g. around gonad with bacteria - 3/5 hemorrhage in fat/serosa next to cervical ln, thyroid, or pancreas
Integument, musculo-skeletal system	5/10 NS 5/10 with lesions (trauma): - 1/5 fracture - 5/5 hemorrhages in neck (*n =* 1; killed), head (*n =* 2), intra-thoracic (*n =* 1), intra-abdominal (*n =* 1)	9/10 NA 1/10 with lesion - 1/1 epithelial ulceration eyelid with hemorrhage and bacteria (trauma)	8/15 NS 7/15 with lesions (trauma): - 1/7 fracture - 2/7 ruptured skin - 4/7 hemorrhages in neck (*n =* 2), head (*n =* 1; killed), intra-abdominal (*n =* 1)	15/15 NA
Eye	10/10 NS (*n =* 1 missing#)	10/10 NA	14/15 NS (*n =* 1 missing#)	15/15 NA
Brain	9/10 NS 1/10 with lesions: - 1/1 intracranial hemorrhage and around cervical spinal cord (trauma)	10/10 with lesions: - 1/10 severe necrotizing meningoencephalitis cerebrum with perivasculitis and hemorrhages - 2/10 hemorrhage in meninges (*n =* 1) or cerebellum (*n =* 1) - 10/10 hyperemia meninges (*n =* 4), cerebellum (*n =* 3), around hypophysis (*n =* 2), choroid plexus (*n =* 1).	4/15 ND 9/11 NS 2/11 with lesions: - 1/2 hemorrhage (trauma; killed) - 1/2 hyperemia	1/15 NA 4/14 NS 10/14 with lesions: - 7/10 hemorrhages e.g., in cerebrum (*n =* 2), cerebellum (*n =* 2), medulla oblongata (*n =* 1) - 9/10 hyperemia, e.g., in meninges (*n =* 2), cerebrum (*n =* 4) cerebellum (*n =* 1), medulla oblongata (*n =* 1)

*NS, no significant lesions; ND, not determined; NA, Not available*.

*PALS, peri-arterial lymphocytic sheets*.

*#scavenged*.

**Figure 3:**
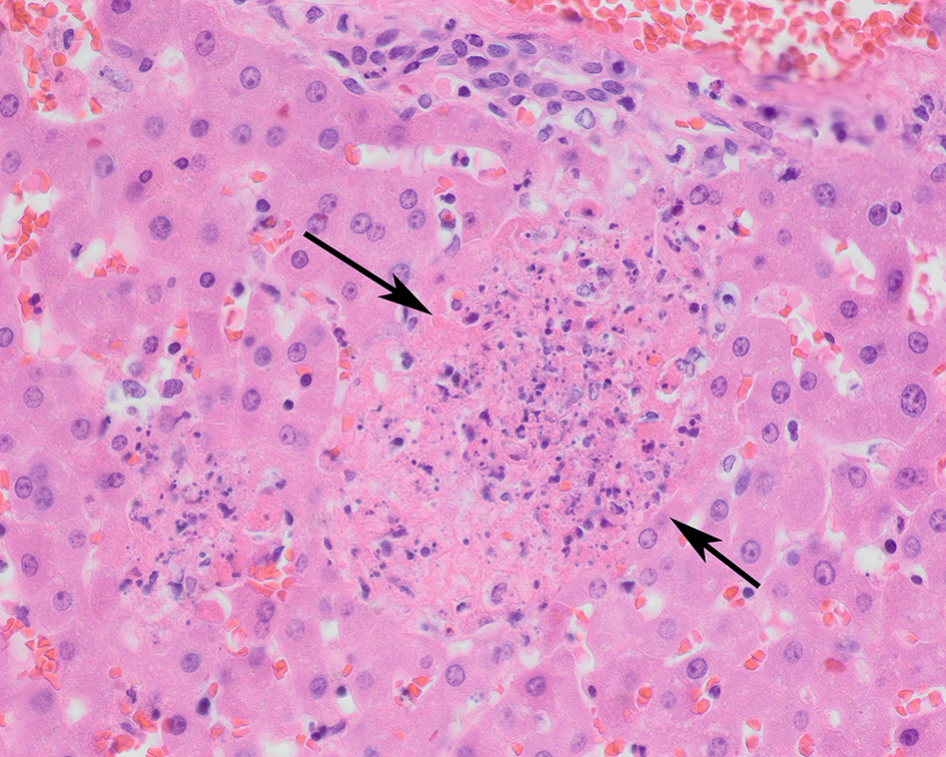
Liver of a tularemia positive hare showing multiple foci of hepatocellular necrosis (arrows). Hematoxylin and eosin stain, magnification x 40.

**Figure 4:**
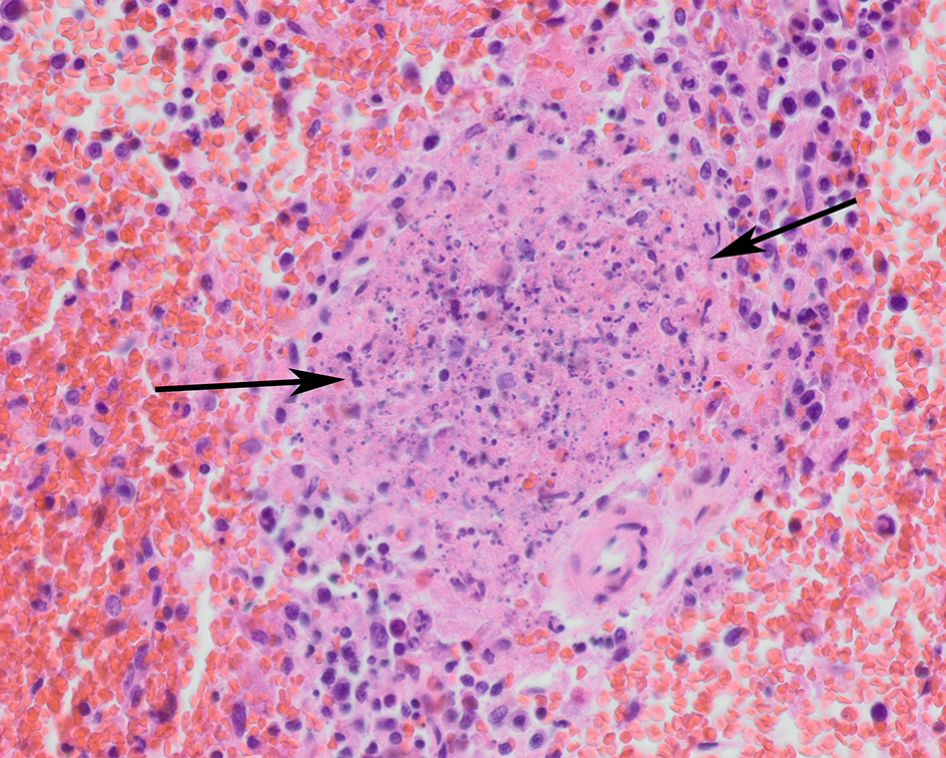
Spleen of a tularemia positive hare showing multiple foci of necrosis (arrows). Hematoxylin and eosin stain, magnification x 40.

Histologically, necrotizing lesions and hemorrhages predominated (Table 3). Severe (sub)acute necrotizing hepatitis and splenitis were the most common lesions. Necrotizing hepatitis was observed in 10/10 (100%) of the B.6 cases and 14/15 (93%) of the B.12 cases (Figure 3). Splenitis was observed in 9/10 (90%) of the B.6 cases and 9/13 (69%) of the B.12 cases (Figure 4). Pneumonia, mostly necrotizing, also commonly occurred in the B.6 cases (6/10, 60%), but rarely in the B.12 cases (2/15, 13%). The odds for the occurrence of pneumonia were significantly greater in the B.6 group than in the B.12 group (Fisher exact test; *p* = 0.02), but this was not the case for hepatitis (Fisher exact test; *p* = 0.60), splenitis (Fisher exact test; *p* = 0.25) or adrenalitis (Fisher exact test; *p* = 0.32). Necrotizing lesions and hemorrhages were observed to a lesser extent in adrenal glands, brain, lymph nodes, and serosa. Necrotizing adrenalitis was observed in 4/9 (44%) of the B.6 cases, and 3/12 (25%) of the B.12 cases. One B.6 case had a severe necrotizing meningoencephalitis. Lymph nodes and serosa were not systematically sampled, but five cases of necrotizing lymphadenitis (three B.6 cases, two B.12 cases) and two cases of B.12 serositis were identified. Multiple organs, also those without necrotizing lesions or hemorrhages, showed hyperemia. Granulomatous lesions were rare (Table 3).

## Discussion and Conclusions

### Genotyping Results and Geographic Distribution

In the Netherlands two different clades of *F. tularensis* subsp. *holarctica*, B.6 and B.12, have been found both in humans and in European brown hares. Strains that could be typed in more detail were found to be members of either B.6 subclade B.11 (five human cases and one hare case) or B.12 subclade B.20 (two human cases and 13 hare cases). One isolate of the B.20 subclade could be further typed as B.33 using the CanSNPer platform. As shown in Figure 2, tularemia is widely dispersed over the country, with cases reported from eight of the 12 Dutch provinces.

In Europe, a clear geographic distribution is obvious for the two main basal clades with the B.12 present in the east of Europe and the B.6 in the west (Gyuranecz et al., 2012; Dwibedi et al., 2016), but regional dispersal occurs also within countries. In Germany, several studies showed that both basal clades are present, with considerable genetic diversity (Schulze et al., 2016). Again, B.12 is found in the east of Germany, and only in a few cases in the north-west of the province of Lower Saxony, while clade B.6 is present in the west of Germany (Muller et al., 2013). In Switzerland both clades are circulating as well, with a predominance of the B.6 subclade B.11 (Dwibedi et al., 2016; Origgi and Pilo, 2016; Wittwer et al., 2018). One B.12 strain in our study was assigned to the B.33 subclade, which has also been reported in Germany, Austria, Hungary and Scandinavia (Gyuranecz et al., 2012; Schulze et al., 2016).

In the Netherlands, the spatiotemporal distribution of the human and hare cases in each basal clade group supports the occurrence of local phylogeographic patterns, as were observed in Sweden (Karlsson et al., 2013). Sequence based data combined with anecdotal information showed that clade B.6 may have been introduced in Europe in the 1930s (Jusatz, 1952; Dwibedi et al., 2016). It is likely that this also included the south of the Netherlands, where the first tularemia cases were reported in 1953. We can only speculate whether *F. tularensis* has been present undetected in the Netherlands in the period in-between or a recent reintroduction has occurred. Indirect support to the former notion comes from recent detection of clade B.12 and clade B.6 in environmental waters in connection with the previously described hare outbreaks in the Netherlands (Janse et al., 2017, 2018). The persistence in water is largely unknown but older literature and more recent research indicate the possibility of prolonged persistence in water courses particularly in endemic regions (Parker et al., 1951; Pomanskaia, 1957; Broman et al., 2011). Thus, it cannot be excluded that the bacterium has been circulating unnoticed in the Netherlands, sustained in the water environment by irregular enzootic amplification cycles in local wildlife or survived during inter-epizootic periods associated with some yet-unknown reservoirs. Alternatively, the current increase in tularemia cases may be the result of a recent reintroduction of the bacterium from neighboring countries.

It is postulated that the persistence and spread of a strain in a certain area, i.e., the occurrence of local phylogeographic patterns, may be associated with strain differences in infectivity and pathogenicity for host species, in combination with ecological differences, i.e., variation in local landscape conditions and the relative abundance of different suitable host vectors (Karlsson et al., 2013; Maurin and Gyuranecz, 2016). This would be an interesting subject for further studies using higher resolution genotyping that also include samples from water and additional wildlife species. Events with grouped hare cases, as observed in Utrecht from May 2016 to May 2017 for the B.6 subclade B.11 and in Friesland from February to May 2015 for the B.12 subclade B.20, will provide good opportunities for this.

### Pathology Findings Per Genotype in European Brown Hares

The European brown hare tularemia cases examined in this study generally had severe (sub-)acute necrotizing lesions and hemorrhages in multiple organs. The distribution of the lesions is consistent with disseminated disease or septic shock. In other studies of tularemia in European brown hares, the association of *F. tularensis* with lesions was confirmed through culture, PCR-tests and immunohistochemistry of multiple organs (Gyuranecz et al., 2010; Origgi and Pilo, 2016; Hestvik et al., 2017). In this study, diagnostic PCR-tests were performed on lung, liver and spleen tissue and in all cases these three tissues were highly positive (data not shown), which is consistent with disseminated *F. tularensis* infection. The disease course was probably rapid because, unlike in other countries with surveillance programs (Gyuranecz et al., 2010; Origgi and Pilo, 2016; Hestvik et al., 2017), granulomatous and necrotizing granulomatous lesions were rare. The pathology findings support high susceptibility of the affected hares to the disease.

Genotype-associated differences in pathology in the European brown hare have previously been suggested and discussed in the light of pathogenicity and route of infection (Origgi and Pilo, 2016). In the Netherlands, so far there is no evidence for a difference in pathology between the two genotypes present in hares, except for a significantly higher number of pneumonia cases in the B.6 compared to the B.12 clade. There could be a trend toward more necrotizing lesions in the B.6 compared to the B.12 cases, but more data are required to substantiate this.

Hemorrhages were a prominent finding in hare cases. Some of these could be attributed to trauma, which was observed in five B.6 and seven B.12 cases. It is likely that the unusual behavior of the hare promoted the occurrence of scavenger attacks or even self-inflicted trauma, because only a fraction of the traumatic lesions corresponded to lesions consistent with the hare being put down for humane reasons by the submitter. Other hemorrhages, often visible only microscopically, were most likely *F. tularensis* disease-related lesions. The occurrence of hemorrhages in European brown hare tularemia cases has been previously described (Origgi and Pilo, 2016).

## Conclusion

This study shows that *F. tularensis* subsp. *holarctica* is endemic and widely distributed in the Netherlands. It is represented by the two basal clades present in Europe, with only slight differences in pathology in European brown hares that were found dead. The main lesions observed in hares for both B.6 cases and B.12 cases were severe (sub-)acute necrotizing hepatitis and splenitis as well as pneumonia in B.6 cases.

The results reported in this study greatly benefitted from the close collaboration between medical, veterinary and wildlife professionals in surveillance, communication and source tracing of *F. tularensis* cases. Continuation of this One Health approach will help improve insight in how the genetic diversity and phylogeny of *F. tularensis* evolves in the Netherlands and in Europe.

## Author Contributions

JR and MiK equally attributed to the writing of the manuscript. AG, H-JR, JR, MiK, and MM contributed to conception and design of the study. JI and MaK performed the post-mortem examinations. DN, ME, MiK, RD, HeB, HaB, SS, and RP were involved in the diagnostic testing. KM, MF, MG, and RR were involved in the genotyping. JR and MaK summarized the pathological findings. EF, JR, MiK, PT, MM, MS, MV, and RP organized and managed the information in relevant databases. JR constructed Figure 2. All authors contributed to manuscript revision, read and approved the submitted version.

### Conflict of Interest Statement

The authors declare that the research was conducted in the absence of any commercial or financial relationships that could be construed as a potential conflict of interest.
